# Room Temperature Ammonia Gas Sensor Based on p-Type-like V_2_O_5_ Nanosheets towards Food Spoilage Monitoring

**DOI:** 10.3390/nano13010146

**Published:** 2022-12-28

**Authors:** Lai Van Duy, To Thi Nguyet, Dang Thi Thanh Le, Nguyen Van Duy, Hugo Nguyen, Franco Biasioli, Matteo Tonezzer, Corrado Di Natale, Nguyen Duc Hoa

**Affiliations:** 1 International Training Institute for Materials Science (ITIMS), Hanoi University of Science and Technology (HUST), No. 1, Dai Co Viet Street, Hanoi 10999, Vietnam; 2 Department of Electronic Engineering, University of Rome Tor Vergata, Via del Politecnico 1, 00133 Rome, Italy; 3 Department of Food Quality and Nutrition, Research and Innovation Centre, Fondazione Edmund Mach, 38010 San Michele All’Adige, Italy; 4 Department of Materials Science and Engineering, Division of Microsystems Technology, Uppsala University, Lägerhyddsvägen, 1751 21 Uppsala, Sweden; 5 Department of Chemical and Geological Sciences, Università di Cagliari, Campus di Monserrato, 09042 Monserrato, Italy; 6 Center Agriculture Food Environment, University of Trento/Fondazione Edmund Mach, Via E. Mach 1, 38010 San Michele All’Adige, Italy

**Keywords:** gas sensor, vanadium pentoxide, ammonia, nanosheet, room temperature, food quality

## Abstract

Gas sensors play an important role in many areas of human life, including the monitoring of production processes, occupational safety, food quality assessment, and air pollution monitoring. Therefore, the need for gas sensors to monitor hazardous gases, such as ammonia, at low operating temperatures has become increasingly important in many fields. Sensitivity, selectivity, low cost, and ease of production are crucial characteristics for creating a capillary network of sensors for the protection of the environment and human health. However, developing gas sensors that are not only efficient but also small and inexpensive and therefore integrable into everyday life is a difficult challenge. In this paper, we report on a resistive sensor for ammonia detection based on thin V_2_O_5_ nanosheets operating at room temperature. The small thickness and porosity of the V_2_O_5_ nanosheets give the sensors good performance for sensing ammonia at room temperature (RT), with a relative change of resistance of 9.4% to 5 ppm ammonia (NH_3_) and an estimated detection limit of 0.4 ppm. The sensor is selective with respect to the seven interferents tested; it is repeatable and stable over the long term (four months). Although V_2_O_5_ is generally an n-type semiconductor, in this case the nanosheets show a p-type semiconductor behavior, and thus a possible sensing mechanism is proposed. The device’s performance, along with its size, low cost, and low power consumption, makes it a good candidate for monitoring freshness and spoilage along the food supply chain.

## 1. Introduction

Ammonia sensors are widely requested in different application areas, such as automotive, environmental monitoring, industrial processes, medicine, agricultural processes (fertilizer, feed), and food [1,2]. Ammonia is not a greenhouse gas, but it can indirectly contribute to greenhouse gas emissions, which can cause serious harm to human health. According to the American Conference of Governmental Industrial Hygienists (ACGIH) and the US Occupational Safety and Health Administration (OSHA), the threshold limit value (TLV) for NH_3_ exposure is 25 ppm [3,4]. 

Ammonia in human breath is produced by the metabolism of protein, and is related to the ammonia produced in the body in various organs and processes [5,6]. For these reasons, it is indicated as a biomarker for specific diseases and conditions, such as renal failure [7], halitosis [8], hepatic encephalopathy [9], and Helicobacter pylori [10]. In exhaled breath, the concentration of NH_3_ reaches 15 and 1.5 ppm, respectively, for end-stage renal disease patients and kidney patients, whereas its concentration is approximately 0.8 ppm in healthy individuals [11].

Ammonia is also among the main indicators of bacterial degradation of protein-rich food (fish, poultry, and meat) [12]. For instance, the concentration of NH_3_ produced during pork decomposition ranges from 10 to 50 ppm [13]. Monitoring of NH_3_ emissions from food would allow early degradation of food to be detected and establish safe consumption. As a result, the development of sensors for NH_3_ detection with high sensitivity, selectivity, speed of response, and low detection limit at room temperature (RT) is crucial [13,14,15,16,17].

Resistive sensors are among the most common gas sensors; they are simple, cheap, fast, sensitive, and stable, and are therefore widely investigated for food analysis. Nanostructured semiconducting metal oxides (SMOs) have gained great attention due to their above-mentioned properties. Nanostructures of metal oxides such as SnO_2_, ZnO, TiO_2_, In_2_O_3_, Fe_2_O_3_, WO_3_, CuO, NiO, MoO_3_, and V_2_O_5_ with different morphologies have been prepared by different methods and used as gas sensors [18,19]. Among them, vanadium oxide is attracting considerable attention because of its multivalent phases and layered structure. Vanadium pentoxide (V_2_O_5_) is an n-type semiconductor with a bandgap between 2.04 and 2.8 eV. It shows high stability, variable oxidation states, high specific capacitance, high energy density, resistivity, and excellent electrical properties [15,16]. Different morphologies of V_2_O_5_ such as nanoparticles, nanowires, thin films, nanospheres, nanorods, nanofibers, and others, have been tested as sensors for many gases such as hydrogen (H_2_), ethanol (C_2_H_5_OH), NO_x_, and ammonia.

Nanomaterials with a porous structure can be easily prepared using inexpensive hydrothermal methods with environmentally friendly reagents. In particular, V_2_O_5_ nanostructures are characterized by high porosity and high surface-to-volume ratio that promote the diffusion of gas molecules inside the sensor material, improving the utilization rate of the sensor and its gas sensitivity [20].

Mounasamy et al. [21] proposed a non-invasive wearable health monitoring device based on V_2_O_5_ nanosheets for ammonia detection. Surface-modified poly-l-lactic acid (PLLA) substrates were used to improve the sensing performance of the V_2_O_5_ film. The sensing mechanism of the PLLA/V_2_O_5_ film was aided by the large specific surface formed by the molding process. The adsorption properties of V_2_O_5_ were exploited to develop ammonia nanogravimetric sensors consisting of V_2_O_5_ nanoplatelets deposited on a QCM piezoelectric transducer [22]. The physical adsorption mechanism and the unique porous structure of the V_2_O_5_ material were used to explain the detection performance towards ammonia. The rough and hydrophilic surface of the V_2_O_5_ thin film promotes the synthesis of additional adsorbed species, increasing the production of ammonium hydroxide (NH_4_OH) and hence the sensitivity of the sensor.

In this paper, we further investigated the sensitivity of V_2_O_5_ to ammonia by testing a V_2_O_5_ nanosheets-based resistive gas sensor obtained using the hydrothermal technique followed by calcination. In addition to being sensitive, the sensor showed good selectivity with respect to seven possible interferers. Despite the known n character of V_2_O_5_, the response to ammonia (an electron donor) is compatible with p-type behavior. It is known that a p-type material can result under particular growth conditions, such as hydrated amorphous V_2_O_5_ [23]. Here, the inversion of the conductivity character is compatible with the formation of a surface inversion layer induced by molecular surface adsorption.

## 2. Materials and Methods

The following chemicals were used: ammonium metavanadate (NH_4_VO_3_, 99.99%), Poly (ethylene glycol)-block-poly (propylene glycol)-block-poly (ethylene glycol) (Pluronic P-123, HO(CH_2_CH_2_O)_20_(CH_2_CH(CH_3_)O)_70_(CH_2_CH_2_O)_20_H, 99%), ethylene glycol (C_2_H_6_O_2_, 99%), and ethanol (CH_3_CH_2_OH, 99.8%) were bought from Sigma-Aldrich (St. Louis, MO, USA). All reagents were analytic grade and used as received without further purification. Deionized water was used as a solvent to prepare the solution. The V_2_O_5_ nanosheets were synthesized using the hydrothermal method. In a typical procedure, ammonium metavanadate (NH_4_VO_3_) (10 mmol) was dissolved in 30 mL of deionized water and 30 mL of ethylene glycol and stirred for 15 min. Then, Pluronic P-123 was added and stirred for 30 min. After that, oxalic acid (C_2_H_2_O_4_) (10 mmol) was added and stirred for another 15 minutes until pH 4 was obtained. The obtained solution was transferred to a 100 mL Teflon-lined stainless-steel autoclave for the hydrothermal process and was maintained at 200 °C for 24 h. After being gradually cooled to room temperature, the precipitate at the bottom was centrifuged and washed several times with deionized water. It was then washed twice with ethanol and collected by centrifugation at 4000 rpm. The obtained product was dried in an oven at 60 °C for 24 h and finally calcined at 500 °C for 2 h.

The structure, morphology, and composition of the synthesized material were characterized using field-emission scanning electron microscopy (FESEM, Hitachi S-4800, Tokyo, Japan), high-resolution transmission electron microscopy (HRTEM, JEM 2100, JEOL Ltd., Tokyo, Japan), X-ray diffraction (XRD, X-Pert Pro, Malvern Panalytical Ltd., Malvern, UK) with a Cu-*K_α_* source in a 2θ range from 10° to 80°, energy-dispersive X-ray spectroscopy (EDS, 7395H, HORIBA, Minami-ku Kyoto, Japan), Raman spectroscopy (Renishaw, InVia confocal micro-Raman Spectroscope, Renishaw, UK), and thermogravimetric analysis (TGA 550, TA Instruments, New Castle, DE, USA). Specific surface area was estimated through BET analysis (Micromeritics Gemini VII 2390, Micromeritics Instrument Corporation, Narcross, GA, USA). The oxidation levels of the material were investigated using X-ray photoelectron spectroscopy (XPS, ULVAC-PHI, 370 Enzo, Kanagawa, Japan).

**Figure 1:**
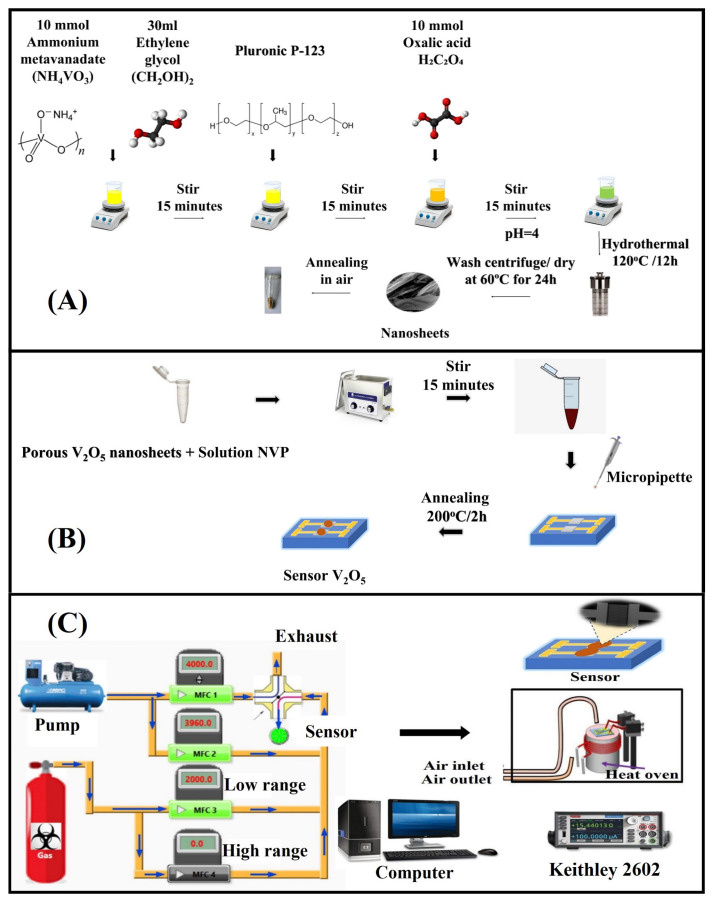
Process diagram of (**A**) hydrothermal synthesis of V_2_O_5_ nanosheets, (**B**) drop coating of the nanomaterial on electrodes to make the gas sensor, and (**C**) homemade gas measurement system.

The gas sensors were prepared, as shown in Figure 1, using drop-casting and following a method found in the literature [24]. The V_2_O_5_ nanosheets were dispersed in N-vinylpyrrolidone to form a colloidal solution and then deposited on a silicon dioxide substrate. The substrate was endowed with a pair of interdigitated comb-shaped Pt electrodes was fabricated by UV photolithography.

The sensing properties were tested using a homemade gas test station. Prior to the measurements, the sensors were preheated at 500 °C for 2 h to stabilize the resistance and enhance the contact between the Pt electrodes and the vanadium pentoxide. The sensor resistance was recorded with a source meter (Keithley 2602, Keithley, Solon, OH, USA) as the atmosphere in the measuring chamber changed from air to analyte gas, and vice versa. The limit of detection was calculated using the sensitivity at the origin and the signal-to-noise ratio of the current signal [25].

## 3. Results and Discussion

### 3.1. Morphological, Compositional, and Structural Characterization

**Figure 2:**
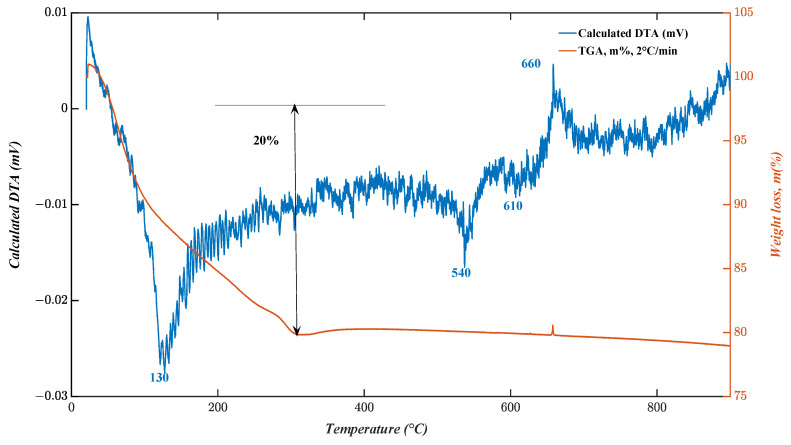
TGA–DTG curves of V_2_O_5_ nanosheets.

Thermogravimetric (TGA) and differential thermal measurements (DTA) were performed in a N_2_ atmosphere at temperatures ranging from 20 to 950 °C to determine both the precursor breakdown temperature and the most suitable calcination temperature required to generate the V_2_O_5_ phase. The results are shown in Figure 2, where two distinct phases are identified.

The modest weight loss is probably due to moisture evaporation. At 320 °C, the TGA curve shows a large weight loss of 20%, which corresponds to the small sharp endothermic peak at 130 °C in the DTA curve. The endothermic peak and the related weight loss are consistent with the combustion of the organic polymer component (Pluronic P123). The formation of V–O bonds has been reported to occur at temperatures above 330 °C [26]. The V–O bonds are supposed to act as crystallization centers for different crystalline vanadium oxides. At temperatures above 550 °C, no further weight loss was found since all organic material had already been destroyed at this temperature. The following increase in mass at 660 °C is most likely caused by the emergence of orthorhombic V_2_O_5_ [27].

**Figure 3:**
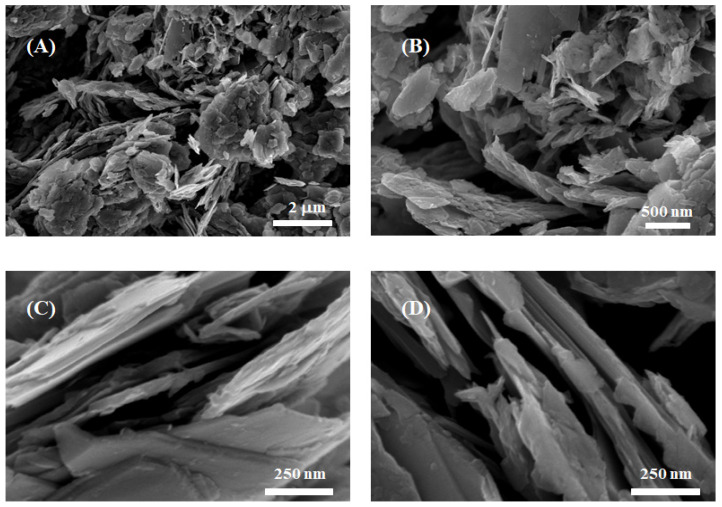
SEM images of the grown nanostructures: (**A**) low magnification; (**B**) medium magnification; (**C**,**D**) high magnification.

The morphology of the growth material was investigated using scanning electron microscopy (SEM). The SEM images in Figure 3 show low, medium, and high magnification and illustrate the uniform presence of wide and thin nanosheets tightly stacked on top of each other. The high magnification images (Figure 3C,D) show an average thickness of approximately 50–60 nm. The large surface area and the small thickness are attributed to the difference in growth rates along different crystal orientations. The formation of stacked V_2_O_5_ thin nanosheets during the hydrothermal process can be described by the following reactions:

(1) $${\mathrm{NH}}_{4}{\mathrm{VO}}_{3}↔{\mathrm{NH}}_{4}^{+}+{\mathrm{VO}}_{3}^{-}$$

(2) $$2{\mathrm{VO}}_{3}+H_{2}↔V_{2}O_{5}+H_{2}O$$

In detail, NH_4_VO_3_, a precursor of vanadium, tends to dissociate into NH_4_^+^ and VO_3_^−^ ions in deionized water [28]. During synthesis, the production of oxalic acid increases the concentration of H^+^ ions that, by reacting with the VO_3_^−^ species, lead to the formation of V_2_O_5_. The addition of the surfactant P123 and the control of the pH value at 5 allow optimization of the formation of thin nanosheets. The resulting nanostructures are characterized by numerous active surface sites that can accelerate the adsorption of target gases.

**Figure 4:**
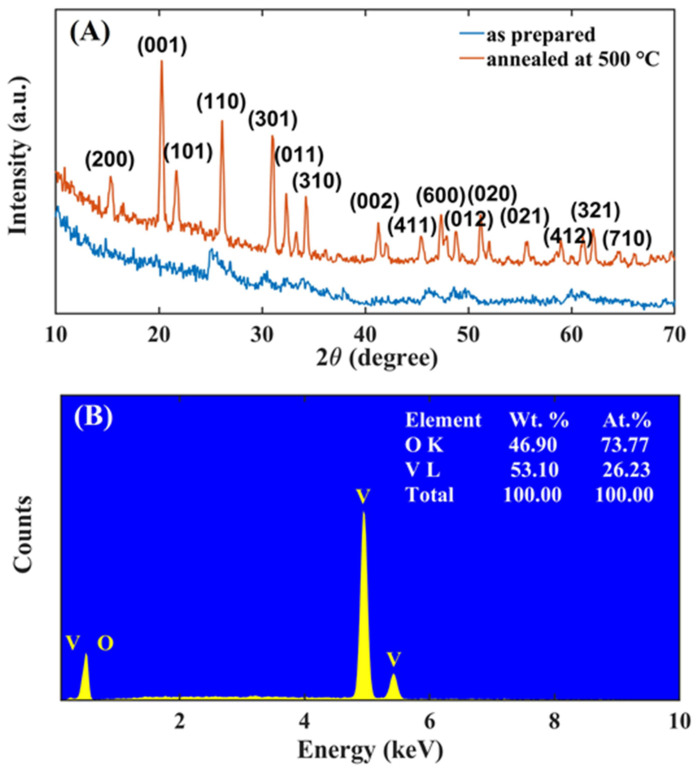
(**A**) XRD spectra of as-prepared V_2_O_5_ nanosheets (blue) and of those annealed at 500 °C (red). (**B**) EDS spectrum.

The structure, purity, and crystalline phase of the nanostructures were investigated using X-ray diffraction analysis. The XRD diffraction patterns of the V_2_O_5_ nanomaterial, unannealed and annealed at 500 °C, are shown in Figure 4A.

Before thermal treatment, the material shows no evident peaks, demonstrating that hydrothermal growth itself produces amorphous nanosheets. Conversely, the spectrum of the nanosheets after calcination at 500 °C (red line in Figure 4A) shows peaks at 2θ = 15.41°, 20.11°, 21.78°, 26.02°, 30.98°, 34.28°, which correspond, respectively, to the lattice planes (200), (001), (101), (110), (301), and (310) of the orthorhombic structure of V_2_O_5_. The diffraction peaks are in good agreement with reference data (JCPDS 41-1426) [29,30,31]. The lack of additional peaks indicates that neither contaminants nor amorphous phases affect the purity of the material [32]. XRD analysis confirms that calcination at 500 °C is crucial to transform the hydrothermally grown material into crystalline V_2_O_5_ nanosheets. The crystallite size of V_2_O_5_ was determined using the Debye–Scherrer equation:

(3) $$D=\frac{k\cdot \lambda }{\beta \cdot \cos\vartheta \;}$$

where *D* is the crystallite size, *k* is the Scherrer constant (approximately 0.89), *λ* is the wavelength of the incident radiation, *β* is the FWHM of the peak, and *ϑ* is the Bragg diffraction angle (*ϑ* = 10°–90°). From the above equation, the average crystallite size in the annealed V_2_O_5_ sample is 32.7 nm. 

The elemental composition of the material was characterized by EDS, the results of which are shown in Figure 4B. The EDS spectrum confirms that the nanosheets are composed only of V and O at weight percentages of 46.90 wt.% and 53.10 wt.%, respectively. EDS does not show additional peaks due to either impurities or contaminants. The atomic ratio between V and O is approximately 2:5, a value compatible with the values in the literature [28].

**Figure 5:**
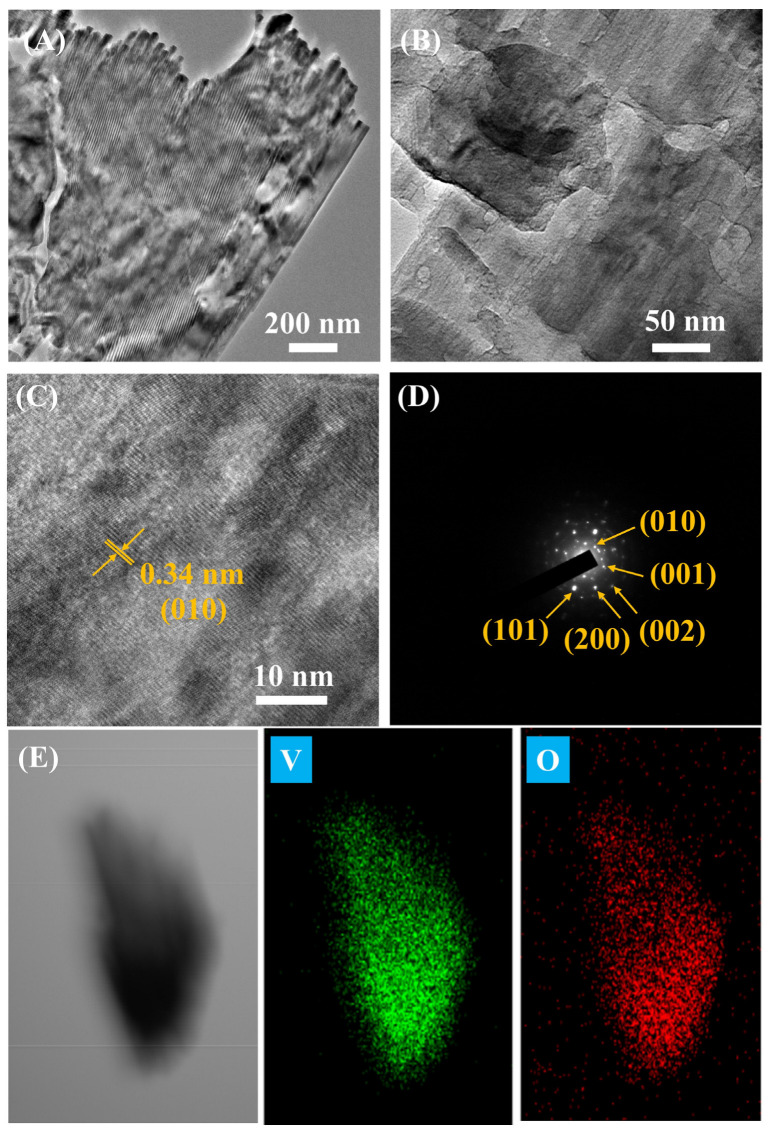
(**A**–**C**) TEM images, (**D**) SEAD, and (**E**) EDS elemental mapping of annealed V_2_O_5_ material. V and O labeled images show the distribution of vanadium (V) and oxygen (O) atoms.

HRTEM images of V_2_O_5_ nanosheets at different magnifications are shown in Figure 5A–C. Crystal lattice fringes are visible in Figure 5C, indicating that the V_2_O_5_ nanosheets have good crystallinity. The lattice spacing at 0.34 nm can be assigned to the (010) plane of the orthorhombic structure of V_2_O_5_ [33,34].

Figure 5D shows the selected area electron diffraction (SAED) pattern. The image shows a sharp and regular pattern of diffraction spots, indicating that the calcined nanosheets are well crystallized. The diffraction points in the crystal correspond to the V_2_O_5_ planes (010), (001), (002), (200), and (101) [35]. The elemental composition maps in Figure 5E show the homogeneous distribution of the elements (V and O).

**Figure 6:**
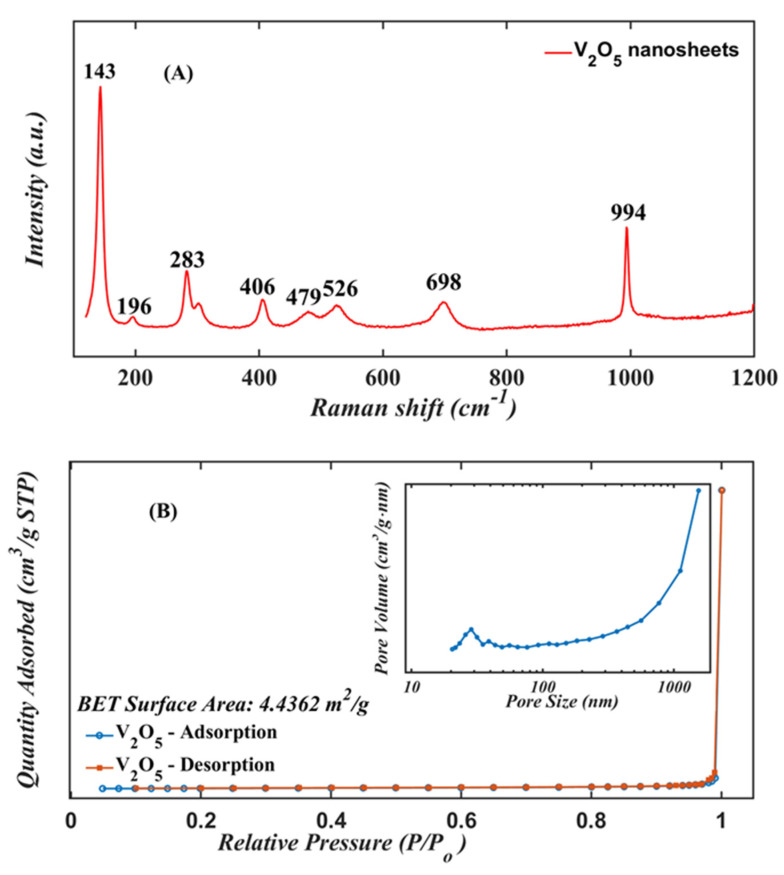
(**A**) Raman scattering and (**B**) BET curve of the V_2_O_5_ nanosheets annealed at 500 °C.

The Raman spectrum of the annealed nanomaterial is shown in Figure 6A. The annealed sample shows peaks at 143, 196, 283, 406, 479, 526, 698, and 994 cm^−1^ [36]. The most intense peak at 143 cm^−1^ corresponds to the vibration of the V–O–V bonds. This peak is evidence of the layered structure of the V_2_O_5_ phase. The peaks at 283 and 406 cm^−1^ correspond to the bending vibrations of the V=O bonds [37]. The peak at 479 cm^−1^ is attributed to the bending vibrations of the V–O–V bonds, whereas the peaks at 526 and 698 cm^−1^ are relative to the phonons band V_3_–O. Finally, the peak at 994 cm^−1^ indicates the terminal oxygen V=O stretching mode, and confirms the crystal quality of the V_2_O_5_ nanosheets [38]. 

Figure 6B shows the nitrogen adsorption/desorption isotherm. The porous structure of the V_2_O_5_ nanosheets is characterized by a specific surface area S_BET_ = 4.4362 m^2^/g. The distribution of pores in the range 20–1000 nm exhibits a peak at about 1000 nm compatible with the presence of multiple nanopores. Because pore size has a direct influence on the diffusion rate of the gas molecules into the sensor layer, the diffusion rate can be estimated from the Knudsen diffusion model:

(4) $$D_{k}=4\frac{r}{3}\sqrt{2\frac{RT}{\pi M}}$$

where *D_k_* is the diffusion rate, *r* is the pore size, *T* is the operating temperature, *M* is the molecular weight of the diffusing gas, and *R* is the universal gas constant. The diffusion rate is proportional to the pore size, so an abundance of pores and/or an increase in their size is expected to enhance gas sensitivity [33].

**Figure 7:**
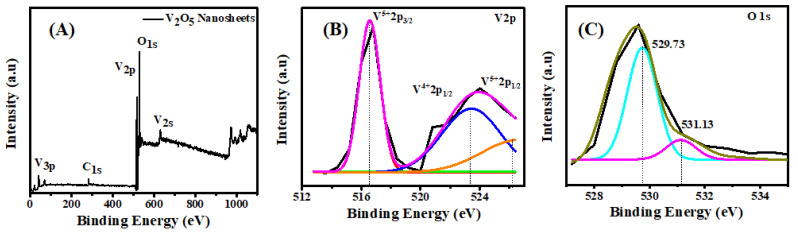
(**A**) Wide binding energy, (**B**) core level V_2p_, and (**C**) core level O_1s_ of XPS spectrums of V_2_O_5_ nanosheets.

The V_2_O_5_ nanosheets were analyzed with XPS to understand their composition and chemical states, and the results are shown in Figure 7. The binding energy spectrum of V_2_O_5_ is depicted in Figure 7A, where the main peaks related to vanadium and oxygen are visible, as well as the sub-peak from carbon at 283 eV. The V_2p_ and O_1s_ core levels of the XPS spectrum are shown in Figure 7B and 7C, respectively. In Figure 7B, there are two broad peaks centered at 516.5 eV and 523.3 eV, which are associated to V2_p3/2_ and V2_p1/2_ doublets and confirm the V^5+^ oxidation state [34]. The binding energy difference of approximately 7.8 eV between V2_p3/2_ and V2_p1/2_ confirms the formation of the orthorhombic structure. The core level of O_1s_ spectrum (Figure 7C) splits into two peaks located at 529.73 and 531.13 eV, which are attributed to the oxygen species O^2−^ adsorbed on the surface of the sensing material. In particular, the peak at 529.73 eV corresponds to the binding of the O and V elements.

### 3.2. Electrical and Gas Sensing Characteristics 

**Figure 8:**
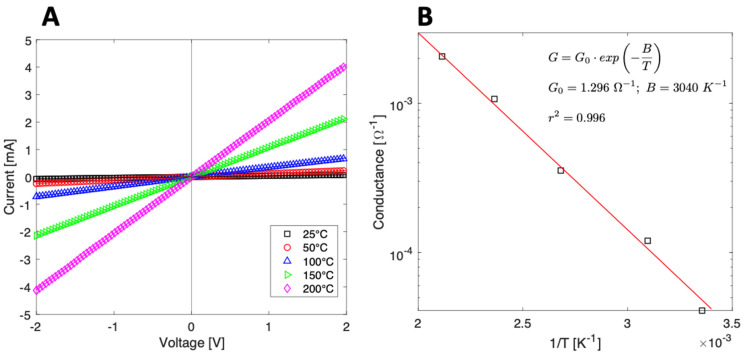
(**A**) I–V curves from V_2_O_5_ nanosheets measured at different working temperatures; (**B**) Arrhenius plot: conductance vs. the inverse of temperature.

The electrical properties of the sensor were analyzed by measuring the *I–V* curves in the range of −2 V to 2 V. Figure 8A shows the I–V curves in nitrogen at different temperatures, from room temperature to 300 °C. The linearity of the *I–V* curves indicates good ohmic contact between V_2_O_5_ and the Pt electrodes. Figure 8B shows the Arrhenius plot of the conductance, where the material exhibits the typical negative temperature coefficient of semiconductors. The relationship between conductance and temperature is defined by an activation energy (*E_a_*), i.e., the minimum energy required to promote the charge carriers [39]:

(5) $$\ln\left(G\right)=-\frac{E_{a}}{k_{B}}\cdot \frac{1}{T}+\ln\left(G_{0}\right)$$

where *G* is the conductance, *k_B_* is the Boltzmann constant, and *T* is the temperature. The fit in Figure 8B estimates an activation energy of 0.26 ± 0.01 eV, which is compatible with band gap values in the literature [22]. 

**Figure 9:**
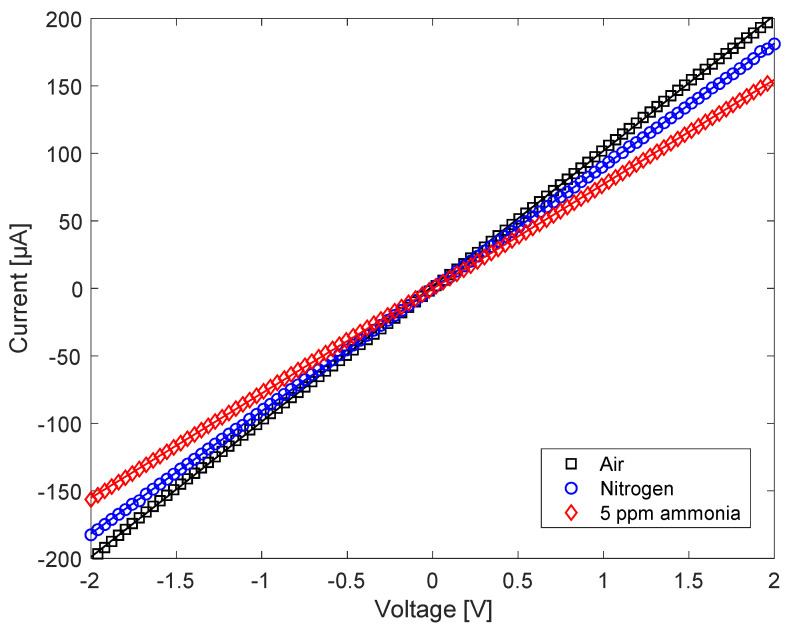
I–V curves of the V_2_O_5_ nanosheets in air, in pure nitrogen, and with 5 ppm of ammonia in air.

**Figure 10:**
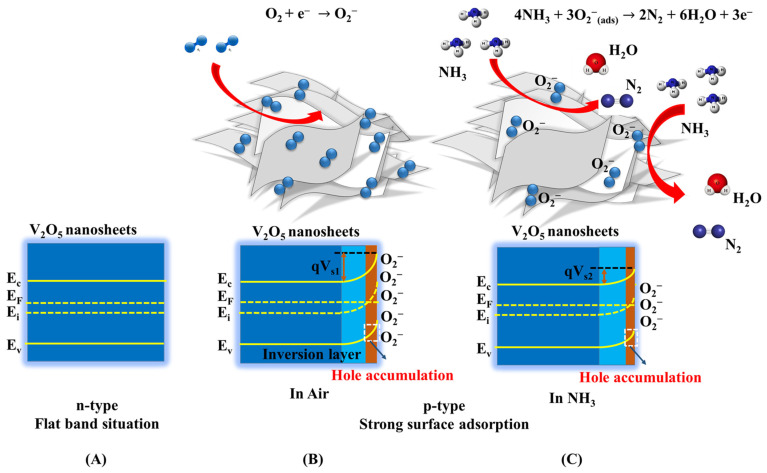
Schematic diagrams of the energy bands of V_2_O_5_ nanosheets in vacuum (**A**); with strong surface adsorption of oxygen in air (**B**); and with ammonia injection (**C**).

The gas effects were initially studied by measuring, at room temperature, the I–V curves of V_2_O_5_ exposed to air, nitrogen, and 5 ppm ammonia in nitrogen. The three curves are shown in Figure 9, and they show that the conductivity decreases from air to nitrogen and even more by adding ammonia to air. Considering the electron acceptor character of oxygen and water vapor and the electron donor character of ammonia, this behavior is compatible with that of a p-type semiconductor. This result is surprising because V_2_O_5_ is commonly known to be an n-type semiconductor. The change in conductivity character has previously been found in hydrated amorphous materials [26], but in this case it seems to be induced by gas adsorption. A thorough explanation of the character change is beyond the scope of this paper and will require more in-depth experimental and theoretical studies. Here, we may assume that this might be caused by the surface depletion of electrons. The mechanism is outlined in Figure 10. When the sensor is exposed to air, the adsorbed oxygen and water molecules are expected to act as electron acceptors, and thus produce an upward band bending at the surface, corresponding to an electron-depleted but hole-enriched region (Figure 10B). The conductivity, on the other hand, increases relative to nitrogen, as expected in a p-type semiconductor. 

In presence of ammonia, the NH_3_ molecules might react with O_2_^−^ on the surface (as in reaction 6), releasing electrons in the material and leading to the decrease of the hole concentration through electron-hole recombination:

(6) $$4{\mathrm{NH}}_{3}+3O_{2}^{-}↔2N_{2}+6H_{2}O+3e^{-}$$

Thus, with the injection of NH_3_, the density of absorbed O_2_^−^ decreases, the energy band bends down, as in Figure 10C, and the sensor resistance increases. This phenomenon is emphasized in a nanometric material where the surface dominates over the bulk.

In most metal oxide semiconductor gas sensors, sensitivity is activated at high temperatures, with the obvious drawback of high-power consumption and the necessity to maintain the sensor temperature stable. Instead, V_2_O_5_ nanosheets have the advantage of being sensitive at room temperature. The sensor sensitivity to ammonia was tested at room temperature and 45% relative humidity. The sensor was exposed to ammonia concentrations ranging from 5 to 500 ppm in air. Each injection of ammonia lasted for 200 s and was followed by exposure to pure air for 800 s to recover the initial baseline.

**Figure 11:**
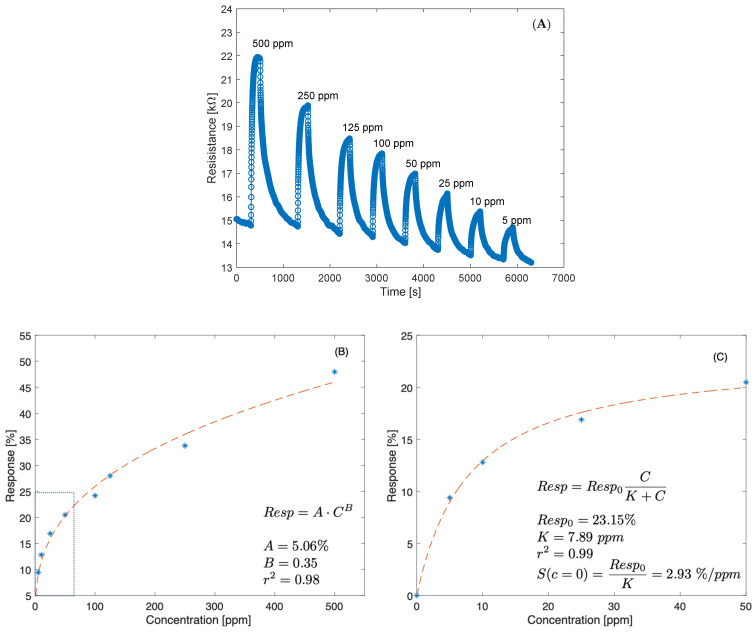
(**A**) Dynamic resistance of the sensor at room temperature and 45% relative humidity towards different NH_3_ concentrations. (**B**) Sensor response curve as a function of the ammonia concentration. Freundlich isotherm fit is also shown (red dashed line). (**C**) Sensor response in the range 0–50 ppm of ammonia fitted by a Langmuir isotherm function. The sensitivity at the origin is calculated as the derivative of the Langmuir function in c = 0 ppm.

The dynamic resistance of the sensor in response to a pulse sequence of ammonia of different concentrations is shown in Figure 11A. As previously mentioned, resistance increases with ammonia concentration. Figure 11B shows the sensor response versus ammonia concentration. The sensor response is calculated as the relative change in resistance:

(7) $$\frac{R_{g}-R_{a}}{R_{a}}\cdot 100\%$$

where *R_a_* and *R_g_* are the resistance in air and in ammonia, respectively. The response curve was fitted with the Freundlich power-law isotherm (Figure 11B). The Freundlich isotherm fits the response over the entire concentration range. However, since the slope (sensitivity) of the Freundlich isotherm at the origin is infinite, it is not adequate to represent sensor behavior in the low concentration range. The response at concentrations below 50 ppm is better fitted by the Langmuir isotherm (see Figure 11C). The deviation at a high concentration from the Langmuir behavior suggests the presence of a low density of high affinity adsorption sites.

The limit of detection for ammonia was calculated using the signal-to-noise ratio and sensitivity at the origin. The sensitivity at the origin, estimated from the Langmuir function, is approximately 2.9%/ppm. The signal-to-noise ratio was calculated in steady-state conditions as the ratio of the average current divided by three times its standard deviation. The measured signal-to-noise ratio is of the order of 50. Thus, the estimated limit of detection is of the order to 0.4 ppm, sufficient for most food quality monitoring applications.

**Figure 12:**
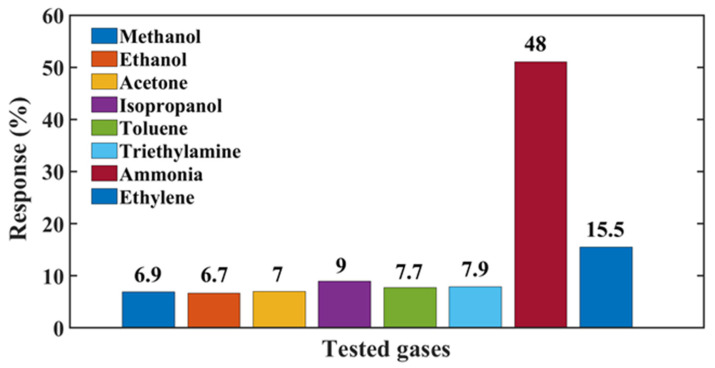
Comparison of the responses of the sensor to various gases.

In terms of practical applications (for example, in the detection of spoilage of meats or fish), selectivity plays a fundamental role in the correct interpretation of sensor signals. The sensor was exposed to 500 ppm of NH_3_ to study its selectivity against 500 ppm of the following interfering compounds: trimethylamine, toluene, isopropyl alcohol, acetone, ethanol, methanol, and ethylene. Sensor responses to the different gases are compared in Figure 12. The change in resistance for NH_3_ is three times larger than for ethylene, and between 5 and 7 times larger than for the other volatile compounds. It is worth mentioning that in most applications, the ethylene concentration is much lower than 500 ppm. The selectivity behavior suggests a high affinity between ammonia and the active sites of V_2_O_5_ [40].

**Figure 13:**
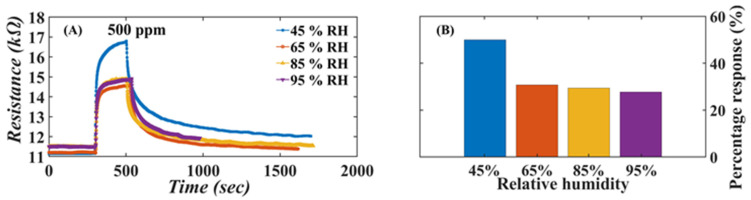
(**A**) Dynamic resistance of the sensor under different humidity conditions; (**B**) sensor response to 500 ppm ammonia under various relative humidity conditions.

Another factor to consider is humidity, which is almost always present, in particular in food and breath. The effect of humidity was tested by measuring the response to 500 ppm ammonia at various levels of relative humidity in the range 45%–95%. The dynamics of the sensor responses are shown in Figure 13A.

Figure 13B shows that the ammonia response is largest at the lowest humidity tested, while at RH larger than 65%, the response to ammonia drops from 50% to 30% but becomes almost insensitive to a further increase in humidity. The decreased response in the presence of humidity suggests that the adsorbed water molecules compete with ammonia for adsorption sites.

**Figure 14:**
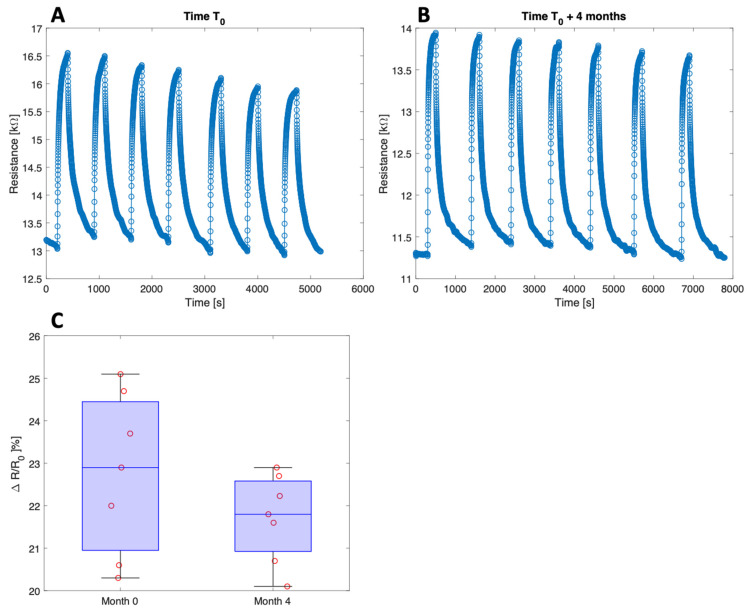
Repeatability and stability tests. (**A**) Sensor dynamic resistance under consecutive exposures to 100 ppm ammonia at 45% relative humidity; (**B**) same measurement repeated four months later. (**C**) Comparison of sensor responses.

Other important parameters to ensure sensor accuracy in real-time monitoring are sensor repeatability and stability. The repeatability and stability were evaluated by measuring the sensor response to repeated cycles of 100 ppm ammonia and by repeating the measurement after four months. The results are shown in Figure 14A,B. Despite the drift of the resistance baseline, the response of the sensor is comparable. Figure 14C shows the distributions of the repeated responses at four months of distance. The stability of the sensor response can be appreciated by calculating the ANOVA of the two distributions. The results indicate that the response to the same ammonia concentration remains statistically undistinguishable (*p*-value = 0.22).

A comparison with recent results reported in the scientific literature from chemoresistive sensors based on pristine and composite V_2_O_5_ nanostructures is summarized in Table 1. V_2_O_5_-based resistive sensors, grown by different methods and with different morphologies, have been tested to detect various gases, such as NO_2_, xylene, ethanol, butylamine, acetone, trimethylamine (TMA), and ammonia. Table 1 indicates that vanadium pentoxide-based sensors are mostly used at high temperatures, in the range of 200–300 °C. 

## 4. Conclusions

Nanostructured V_2_O_5_ was prepared using an environmentally friendly hydrothermal method followed by heat treatment at 500 °C in air. Thanks to the morphology of very thin nanosheets stacked on top of each other, the material has a high surface/volume ratio. The V_2_O_5_ nanosheets show p-type semiconductor character, probably due to the growth process and the peculiar morphology. The resistive sensor based on V_2_O_5_ nanosheets works at room temperature, showing high sensitivity and low limit of detection towards ammonia, a typical gas generated, for instance, by bacterial spoilage of food products. The sensor also exhibits high selectivity to ammonia versus interfering gases such as methanol, ethanol, acetone, isopropanol, toluene, trimethylamine, good repeatability, and good long-term stability. The performance of the chemosensor, together with its simplicity and cost-effectiveness, makes it a candidate for real-time monitoring of ammonia, for instance in breath and for assessing food quality along the production and distribution chain.

## Figures and Tables

**Table 1:** Performance comparison of V_2_O_5_ nanostructures as gas sensors in recent scientific literature.

Material	Method	Gases	Conc.	Response	Temp. (°C)	Ref.
SnO_2_@ V_2_O_5_	ALD	NO_2_	100 ppm	2.5 ^a^	250	[41]
V_2_O_5_ flower-like	Hydrothermal	Xylene	100 ppm	2.2 ^b^	300	[34]
V_2_O_5_ nanosphere	Solvothermal	Xylene	100 ppm	2.757 ^b^	290	[42]
V_2_O_5_ nanorods	Hydrothermal	C_2_H_5_OH	3000 ppm	13.3 % ^c^	100	[43]
V_2_O_5_ thin film	Chemical spray	NO_2_	100 ppm	20.3 % ^d^	200	[44]
V_2_O_5_ nanofibers	Spray pyrolysis	Xylene	100 ppm	27 ^b^	RT	[45]
V_2_O_5_@TiO_2_ core-shell	Sol-gel	NH_3_	100 ppm	8 ^b^	365	[46]
V_2_O_5_ hierarchical	Hydrothermal	1-butylamine	100 ppm	2.6 ^b^	140	[47]
MoO_3_-V_2_O_5_	Spray pyrolysis	NO_2_	100 ppm	80 % ^d^	200	[48]
V_2/_O_5_ flower-like	Hydrothermal	TMA	5 ppm	2.25 ^b^	200	[28]
3.5% Au/V_2_O_5_ microflowers	In situ reduction and thermal oxidization	1-butylamine	100 ppm	7.3 ^b^	240	[49]
V_2_O_5_/CuO nanostructures	Electrospinning	Acetone	500 ppm	8.8 ^b^	440	[50]
2 wt% Sn doped V_2_O_5_		NH_3_	50 ppm	77.84% ^c^	RT	[51]
V_2_O_5_/PVP	Electrospinning	NH_3_	0.6 ppm	6% ^e^	260	[52]
V_2_O_5_ Fibers	Sol–gel	NH_3_	2.1 ppm	11% ^e^	200	[53]
V_2_O_5_ Films	RF sputtering	NH_3_	75 ppm	17 ^b^		[21]
V_2_O_5_ nanosheets	Hydrothermal	NH_3_	100 ppm	24.2% ^d^	RT	This work

^a^ Response defined as R_g_/R_a;_^b^ Response defined as R_a_/R_g;_^c^ Response defined as (I_gas_ – I_air_)/I_air_ × 100 (%); ^d^ Response defined as (R_gas_ – R_air_)/R_air_ × 100 (%), ^e^ Response defined as (R_air_ − R_gas_)/R_gas_ × 100 (%).

## Data Availability

Not applicable.
